# Towards the development of day one competences in veterinary behaviour medicine: survey of veterinary professionals experience in companion animal practice in Ireland

**DOI:** 10.1186/s13620-018-0123-3

**Published:** 2018-05-12

**Authors:** Olwen Golden, Alison J. Hanlon

**Affiliations:** 1 Department of Agriculture, Food and the Marine, Central Veterinary Research Laboratory, Backweston, Celbridge, Co. Kildare Ireland; 2 School of Veterinary Medicine, University College Dublin, Dublin, Ireland

**Keywords:** Veterinary behaviour medicine, Companion animal behaviour, Day one competences, Veterinary behaviour curriculum

## Abstract

**Background:**

Veterinary behaviour medicine should be a foundation subject of the veterinary curriculum because of its wide scope of applications to veterinary practice. Private practitioners are likely to be the primary source of information on animal behaviour for most pet owners, however studies indicate that behavioural issues are not frequently discussed during companion animal consultations and many practitioners lack confidence in dealing with behavioural problems, likely due to poor coverage of this subject in veterinary education.

There is a need to identify learning outcomes to support day one competences in veterinary behaviour medicine and these should be informed by practice-based evidence. This study aimed to investigate the nature and frequency of behavioural queries experienced by veterinary professionals in Ireland, the provision of behavioural services at companion animal practices, behaviour referral practices and challenges associated with providing a behaviour service.

**Methods:**

Two online surveys were developed, one for private veterinary practitioners (PVP) and one for veterinary nurses (VN). Invitations to participate were distributed using contact details from the Premises Accreditation Scheme database on the Veterinary Council of Ireland website. Thirty-eight PVPs and 69 VNs completed the survey.

**Results:**

Results indicated that less than half of companion animal practices offer behavioural consults and under a third of practices provide training and socialization events. Over half of the practices surveyed have referred cases to a behavioural specialist.

The majority of respondents encountered behavioural queries weekly. Ninety-eight percent reported receiving queries regarding dog behaviour. Toilet training and unruly behaviour were two issues encountered frequently. Behavioural issues in cats were also common. House soiling and destructive behaviour were the problems most frequently encountered by respondents.

The two most commonly cited barriers to providing behavioural consultations were lack of in-house or personal expertise, and that clients were not willing to pay for these services.

Furthermore over half of all veterinary professionals surveyed indicated that they had received inadequate undergraduate training in veterinary behaviour medicine.

**Conclusions:**

Behavioural problems in companion animals can affect the quality of life of pets and their owners. Our survey findings indicate that many veterinary professionals frequently encounter behavioural problems and identify an opportunity for improved provision in behaviour medicine in veterinary education.

**Electronic supplementary material:**

The online version of this article (10.1186/s13620-018-0123-3) contains supplementary material, which is available to authorized users.

## Background

Veterinary behaviour medicine should be one of the foundation subjects of the veterinary education curriculum because of it’s wide scope of applications to veterinary practice. For example, it is the first clinical sign of pain, injury and disease in animals, and underpins best practice with animal handling [1]. It is also a key indicator of animal welfare. In the companion animal sector, veterinary knowledge of animal behaviour is essential to addressing common behaviour problems in dogs and cats, which can affect the quality of life of the animal and owner.

McGreevy and Bennett [2] have reported a loss of ‘animal sense’ in pet owners, that as society moves towards urbanization we lose touch with what is normal regarding animal behaviour. Private veterinary practitioners (PVP) are likely to be the primary source of information on animal behaviour for most pet owners, however studies have indicated that opportunities to discuss behavioural issues and intervene in behavioural problems during companion animal consultations are often missed or not fully utilized [3, 4]. Inadequate assessment of these behavioural issues in veterinary practice, and timely referral to a specialist when required, have important implications for welfare [3], resulting in an increased risk of surrender of companion animals to shelters or euthanasia [4]. Whilst research has highlighted client demand for behavioural medicine, practitioners feel ill equipped to deal with such cases [3, 5], most likely due to poor coverage of animal behaviour in veterinary education [6, 7].

The European Coordination Committee for Veterinary Training (ECCVT) [8] published revised Day One Competences in veterinary medicine in 2015. They state that new veterinary graduates should have knowledge and understanding of ‘*the structure, function and behaviour of animals*’. In 2011, the Federation of Veterinarians of Europe (FVE) and American Veterinary Medical Association (AVMA) collaborated to produce 12 competences [9], which included: ‘*Adequate clinical experience to diagnose, treat and prevent mental and physical disease, injury or defect in an animal*’. Whilst behavioural medicine is clearly a requirement for these competences, no specific guidance was provided. In the context of the veterinary education curriculum, there is a need to identify learning outcomes to support day one competences in behavioural medicine, which should include practice-based evidence. The aim of this study was to investigate the nature and frequency of behavioural queries experienced by veterinary practitioners and veterinary nurses in Ireland, the behavioural services on offer in companion animal practices, behaviour referral practices and the perception among veterinary professionals of the challenges associated with providing a behaviour service.

## Methods

Two online surveys were created in SurveyMonkey® to explore the type and frequency of animal behaviour problems experienced by private veterinary practitioners (PVP) and veterinary nurses (VN) working in practice. An invitation to participate, which included links to the online surveys, was emailed to veterinary practices listed in the Premises Accreditation Scheme (PAS) database on the Veterinary Council of Ireland (VCI) website [Accessed 17 Feb 2017]. In total, 377 veterinary premises (categorised as Companion Animal Registered Veterinary Hospitals (RVH (CA)) and Registered Veterinary Clinics (RVC)) were emailed via MailChimp®. In the invitation, recipients were requested to circulate the email to all PVPs and VNs in the practice.

The survey was hosted online for 3 weeks from March 28th to April 18th 2017. Two reminder emails were sent to non-responders after the first and the second week respectively. In addition, the Irish Veterinary Nurses Association (IVNA) was contacted and an email containing the link to the VN survey was circulated to all of their members. Twitter alerts were also used to raise awareness of the surveys, requesting PVPs and VNs to email a dedicated University College Dublin address to access the survey. These alerts were sent twice during the three-week period.

The nature of the survey qualified for exemption from full ethical approval by the Human Research Ethics Committee at University College Dublin (LS-E-17-33-Hanlon).

### Survey design

The surveys consisted of 18 questions. Both surveys recorded the same information, except for the wording of questions relating to client interactions and case referrals in order to reflect the different roles of PVPs and VNs. The first two questions recorded the profession and year of graduation of the respondent. They also redirected respondents taking the wrong survey (ie PVPs taking the VN survey and vice versa). Information was requested on practice profile, whether it was rural/urban, species treated, number of veterinary professionals working in the practice, the behavioural services on offer in the practice and the staff members or external specialists responsible for these services.

Questions 10 to 16 related to general behavioural queries in regards to frequency and species, referral practices for behavioural problems and the frequency of specific behavioural issues for each companion animal species (canine, feline, equine). For the latter, participants were provided with a list of behavioural issues (Table 1) and requested to state if they encountered these issues daily, weekly, monthly or less often.

**Table 1:** List of canine, feline and equine behaviour issues included in the survey

Species	Behaviour Problems
Canine	Toilet training Inappropriate elimination Aggression towards people Aggression towards other dogs Dog-reactive (dogs that over-react to other dogs) Anxiety-related problems (e.g. separation anxiety) Fearful behaviour (e.g. fear of fireworks, fear of the hoover) Destructive behaviour (e.g. chewing furniture) Compulsive disorders (e.g. tail-chasing, acral lick dermatitis) Unruly behaviour (e.g. pulling on lead, jumping up) Roaming or escaping Poor recall Vocalisation Other (please specify)
Feline	House soiling Destructive behaviour (e.g. scratching furniture) Aggression towards owner and other family members Aggression towards guests Aggression towards other pets Overgrooming, wool or blanket-sucking Anxiety-related problems (e.g. hiding) Vocalisation Fearful of other cats, dogs or people Other (please specify)
Equine	Handling problems (e.g. headshyness, barging) Riding problems (e.g. bucking, spooking) Stereotypies or stable vices (e.g. crib-biting, weaving)

The final two questions aimed to establish the perception of veterinary professionals on the key challenges to providing behavioural consultations in practice and the experience of veterinary professionals in providing clinical advice on behavioural issues, in terms of the adequacy of their undergraduate training in this discipline.

The penultimate draft of the survey was peer-reviewed by three veterinary professionals with expertise in behavioural medicine in Ireland and amendments made based on their recommendations.

A copy of each survey can be found in the supplementary materials (see Additional files 1 and 2).

## Results

Due to the method of distribution it was not possible to calculate an accurate response rate. The survey was aimed at PVPs and VNs working in companion animal practice. Forty-one PVPs accessed the survey and 38 completed it. One hundred and six VNs accessed the survey and 69 completed it.

### Respondent and practice profiles

**Fig. 1:**
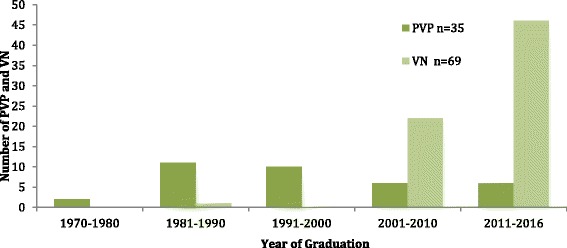
Year of graduation of Private Veterinary Practitioners (PVP) and Veterinary Nurses (VN) survey respondents. PVP: Private Veterinary Practitioners, VN: Veterinary Nurses

The majority of PVP respondents graduated in the 1980s. In contrast, the majority of VN respondents had graduated since 2011 (Fig. 1).

**Fig. 2:**
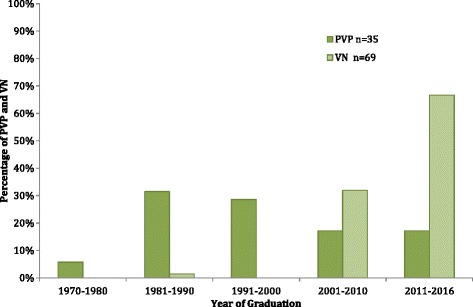
Species treated in the veterinary practices of survey respondents. PVP: Private Veterinary Practitioners, VN: Veterinary Nurses

Over half of the PVPs worked in a rural area, followed by urban and suburban practice. Of the VN respondents, almost 80% worked in either urban or suburban practices. For both respondent populations, the practice size was most commonly 3 PVPs and between 1 and 3 VNs. Dogs and cats were treated in almost all of the practices. Over half of the PVP practices treated horses, compared to 32% of the VN respondent practices (Fig. 2).

### Behavioural services

Of the PVP respondents, 40% of the practices offered behavioural services. Similar results were seen with VN respondents, with 33% of the practices offering behavioural consults. The majority of the behavioural consultations were provided by a PVP without a qualification in behaviour (14 /15 PVP and 9/23 VN responses). The VN respondents (8/23) stated that behavioural services were given by a VN with a behavioural qualification. Six of this group answered that behaviour consultations were conducted by an external specialist who was a veterinary surgeon.

Only 16% of the PVP respondents reported that their practice provided puppy parties, play dates or other training events. In contrast, VN respondents indicated that 30% of their workplaces provided these training events. The majority of VN (16/21) respondents indicated that these training events were run by a VN with no behavioural qualification.

### Type and frequency of behavioural queries

The VN respondents indicated a higher frequency of client queries on behavioural issues (44% daily and 49% weekly) compared to PVPs (16% daily, 46% weekly and 22% monthly).

In both the PVP and VN respondent populations, over 98% stated that they received queries about dog behaviour. Behavioural issues in cats were also common, with 53% of the PVP and 65% of the VN groups reporting that they were asked about cat behaviour. Thirteen percent of the PVP respondents stated that they were asked about horses, compared to only 1% of the VN respondents. Respondents were given the option to provide additional information: 8% of PVPs and 4% of VNs referred to client queries about exotics and small rodents such as rabbits and guinea pigs.

### Behaviour referrals

Fifty-four percent of the PVP respondents reported that they had referred animals to a behaviour specialist and 73% of the VNs worked in a practice where an animal had been referred. In relation to the qualification of the specialist, 62% of PVP respondents had referred to a veterinary surgeon with a specialisation in animal behaviour. Eighteen out of 48 (37%) VN respondents stated that an animal had been referred to a Certified Clinical Animal Behaviourist (CCAB), 23% to a veterinary surgeon with a behaviour specialisation and 31% did not know the qualification of the specialist.

### Nature of behavioural problems encountered in practice

**Fig. 3:**
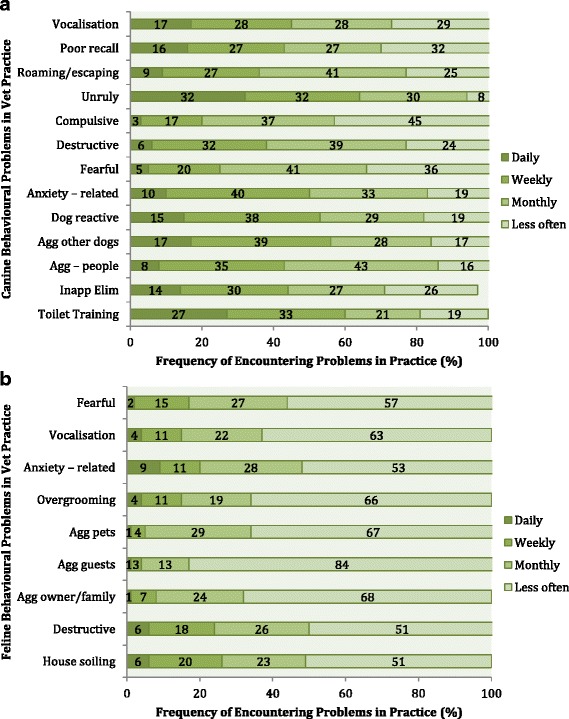
**a** Type and frequency of canine behavioural problems encountered in survey respondents’ veterinary practices. Inapp Elim: Inappropriate elimination. Agg – people: Aggression towards people, Agg other dogs: Aggression towards other dogs. **b** Type and frequency of feline behavioural problems encountered in survey respondents’ veterinary practices. Agg owner/family: Aggression towards owner or family members, Agg guests: Aggression towards guests, Agg pets: Aggression towards other pets

Questions 14–16 related to the types and frequency of behavioural problems encountered by veterinary professionals in practice. Regarding canine behaviour problems, toilet training was a commonly reported issue by both groups. ‘Unruly behaviour’ was a problem that many VN respondents encountered daily, but the PVP respondents did not report it as a common issue (Fig. 3a).

Regarding cat behavioural issues, both groups indicated that they did not encounter many of these issues on a daily or weekly basis. PVP and VN respondents encountered house soiling and destructive behaviour problems frequently. Twelve percent of the VN respondents encountered anxiety-related behaviour daily, however the PVP respondents did not report this as a common problem (Fig. 3b).

Thirteen PVPs answered the question on horse behavioural issues. Although 5 practitioners (38%) stated that they observed handling problems monthly, the vast majority of PVPs saw equine behavioural issues less frequently than monthly. Eleven VNs responded to this question and similarly, the majority encountered problems less often than monthly. Four respondents stated that they encountered stereotypies at a monthly frequency.

### Barriers to providing behavioural consultations

**Fig. 4:**
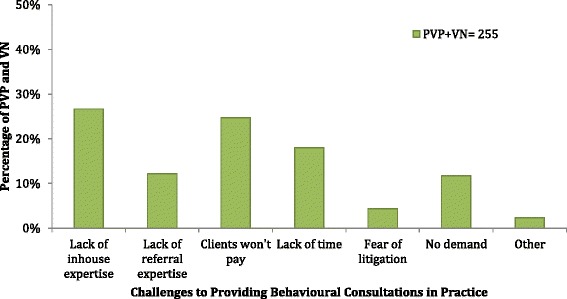
Responses of private veterinary practitioners (PVP) and veterinary nurses (VN) regarding the challenges to providing animal behavioural consultations

Respondents were permitted to select more than one response for this question. Of the 37 PVP respondents, 59% stated that ‘Clients not willing to pay’ was the main obstacle, followed by ‘Lack of in-house or personal expertise’ (57%). Forty-nine percent stated that ‘Lack of time’ was a key factor and 30% felt that there was a lack of referral expertise. Twenty-seven percent selected ‘No demand for animal behaviour consultations’ as an obstacle. Of the 68 VN respondents, 69% felt that ‘Lack of in-house or personal expertise’ was a key factor. Sixty percent indicated ‘Clients not willing to pay’ as a key obstacle and ‘Lack of time’ was selected by 41% of VN respondents (Fig. 4).

### Provision of professional training in animal behaviour

The final question asked respondents to describe their clinical experience of dealing with behavioural issues with regards to undergraduate training (Table 2). Of the 38 PVP respondents, 45% indicated that they had received inadequate training to provide clinical advice on behaviour. Thirty-two percent felt that they had received inadequate undergraduate training but had developed a competency in behaviour. Eighteen percent reported that they had received adequate training but did not see sufficient cases to maintain competency. Only 5% felt that they had received adequate undergraduate training and saw sufficient cases to maintain competency. Of the 69 VN respondents, 32% reported that they had received inadequate training to provide clinical advice on behaviour; 29% that they had received inadequate undergraduate training but had developed a competency in behaviour; 23% indicated that they had received adequate training but did not see sufficient cases to maintain competency. Only 7% reported that they had received adequate undergraduate training and saw sufficient cases to maintain competency. None of the PVP respondents had completed a further qualification in veterinary behaviour, compared to 6 out of 69 VN respondents.

**Table 2:** Clinical experience of Private Veterinary Practitioners (PVPs) and Veterinary Nurses (VN) dealing with animal behavioural issues in practice with regards to undergraduate training

Statement describing clinical experience of dealing with behavioural issues in practice	Number of PVP and VN that picked statement as most accurate description of clinical experience (*n* = 107)	% PVP *n* = 38	% VN *n* = 69
I received inadequate undergraduate training to provide clinical advice on animal behaviour.	39	45%	32%
I received inadequate undergraduate training but have developed a competency in animal behaviour.	32	32%	29%
I received adequate undergraduate training but do not see a sufficient number of cases to maintain competency.	23	18%	23%
I received adequate undergraduate training and I see a sufficient number of cases to maintain competency.	7	5%	7%
I received inadequate undergraduate training but I have obtained a further qualification in veterinary behaviour.	4	0%	6%
I received adequate undergraduate training and I have obtained a further qualification in veterinary behaviour.	2	0%	3%

## Discussion

This survey is part of a broader research aim to develop day one competences for veterinary behaviour medicine, by exploring queries encountered in companion animal practice, what species they related to, the referral policy for behavioural issues and behavioural services offered by veterinary practices. This information will be used to inform teaching practices at the UCD School of Veterinary Medicine. The response rate to this survey cannot be determined and a key challenge with this study was gaining access to veterinary professionals in Ireland due to data protection laws.

The majority of PVP respondents had graduated in the 1980s (range 1970 to 2016). In contrast, except for one VN respondent, the majority had graduated since 2010. Respondents were contacted through email addresses provided on the VCI Premises Accreditation Scheme (PAS), therefore many of the PVPs were likely to be practice principals or partners, which could explain the age profile of the respondents. The age profile of the VN respondents is likely due to the fact that training programmes in veterinary nursing in Ireland commenced in 2000. It is likely that the results for PVPs may have differed if more recent graduates had participated, reflecting the advancement of research underpinning veterinary behaviour medicine. However, the time assigned to animal behaviour in the veterinary curriculum at UCD has not been commensurate with the substantive increase in knowledge and application of veterinary behaviour medicine.

The practice profile of the PVPs had a higher percentage of rural practices, while the VN respondent population was predominantly urban or suburban. This trend is also seen in the responses to the question regarding species treated in the practice. Less cattle and sheep were treated in the practices described by the VN group. This may be a reflection of the lower employment rates of VNs in farm animal practice.

The majority of respondents’ practices do not provide behavioural services such as behavioural consultations and training events. In most cases, these services were provided by a veterinary professional with no specialisation in behaviour, which is a concerning trend. Training events or behavioural consultations provided by staff without specialist knowledge are unlikely to be as effective as those provided by specialists in the area. For example, dominance theory in dog behaviour has been refuted in recent years [10] and applying ill-informed training techniques can be harmful to dog welfare [11]. There is a moderately high rate of referrals in respondents’ practices (54% of PVPs and 73% of VNs). The majority of these referrals were to a veterinary surgeon with a behavioural specialisation. Referrals to a Certified Clinical Animal Behaviourist were also relatively common. It is possible that there is overlap between these two groups.

Regarding the frequency of behaviour queries, while a high proportion of the VN respondents received daily enquiries, for both groups behavioural enquiries were usually a weekly occurrence. Dogs were the species most commonly asked about, followed by cats. There was a marked difference in the incidence of equine queries between the PVP and VN groups, which may reflect VN employment trends in Ireland.

Toilet training was one of the most commonly reported issues, although these may be conversations with a preventative aim, for example brought up at a puppy’s first vaccination consultation, as opposed to a discussion about a behaviour ‘problem’. Unruly behaviour was a common issue reported by VN respondents. It is possible that issues like these may come up more frequently while chatting with clients outside of the consulting room, or that it was observed first hand while dealing with in-patients. Meyer [12] reported that owners were most concerned by unruly behaviour, whereas vets were consulted most about more complex behaviours. In the 2016 People’s Dispensary for Sick Animals (PDSA) animal wellbeing report [13], similar behaviours (jumping up at people) were the most commonly reported issue by dog owners. Both PVP and VN respondents encountered aggression towards other dogs and anxiety-related problems frequently. These are common issues and may be particularly evident in dogs visiting a veterinary clinic and waiting in the reception area with other dogs. In the 2016 PDSA survey, 3% of the dog owners surveyed stated ‘biting other dogs’ as an issue and 5% reported that their dogs showed signs of distress when left alone. Although the latter issue related only to separation anxiety, it is part of the broader spectrum of anxiety-related behaviours. Destructive behaviour and aggression towards people were encountered less frequently by our respondents. Seven percent of dog owners in the PDSA survey reported destructive behaviour as an issue. Aggression towards people was reported by 3% of this group [13].

In cats, house soiling and destructive behaviour were two of the most frequently encountered issues. Destructive behaviour was the most common undesirable behaviour reported by cat owners in the PDSA survey [13]. It was also the most frequently reported behavioural problem in a recent survey of Danish cat owners [14]. In the Danish survey, house soiling was also a common problem reported by owners. Nine percent of the UK cat owners (2, 246 owners) surveyed by the PDSA reported it as an issue [13].

It is interesting to note that the PVP respondents did not report anxiety-related behaviour as a frequent occurrence, but 12% of VN respondents reported that they encountered it daily. Similar to unruly behaviour in dogs, this may be something that VNs discuss with clients outside of the consulting room or encounter with inpatients.

The majority of respondents cited ‘lack of inhouse or personal expertise’ as a barrier to providing behaviour consultations. In a survey of UK veterinarians in small animal practice [3], the majority of PVPs surveyed did not feel confident dealing with behavioural problems. Similar findings were reported in a survey by Patronek and Dodman [6]. When asked to select a statement that best described their experience of dealing with behavioural issues in practice, the majority of veterinary professionals stated that they did not receive adequate undergraduate training to provide advice on animal behaviour. The second most frequent choice also referred to inadequate undergraduate training and the subsequent development of a competency in animal behaviour. This indicates that the undergraduate curriculum in behavioural medicine is failing to prepare veterinary surgeons and veterinary nurses in Ireland for the assessment and treatment of behavioural issues in practice. It is important to note that our respondents, particularly the VNs may have graduated from a number of different educational institutions. A lack of time and resources dedicated to behavioural teaching in veterinary schools has been noted in the UK [7] and the US [6, 15].

The Day One Competences outlined by the ECCVT [8] and the FVE and AVMA [9] include references to knowledge of animal behaviour and an understanding of the diagnosis and treatment of mental disease. However, based on the feedback from veterinary professionals surveyed in our study and of those in other studies [3, 4, 15], there is a need for comprehensive learning outcomes for veterinary behavioural medicine and a clearly defined set of day one competences. Engagement with veterinary professionals, specialists in veterinary behaviour medicine and other stakeholders such as companion animal owners would be of value, to identify Day one skills and knowledge including an understanding of how behavioural issues affect the human-companion animal bond and client expectations regarding the supports they feel veterinary professionals can put in place to deal with these problems or to prevent them.

## Conclusions

Behavioural problems in companion animals can affect the quality of life of pets and their owners and have serious welfare implications. Our survey findings indicate that veterinary professionals frequently encounter queries about animal behaviour, but feel that their undergraduate training did not prepare them for dealing with these problems in practice. Furthermore it highlights an opportunity to develop behavioural medicine within the veterinary education curriculum to facilitate the wider provision of behavioural services in veterinary practice. Whilst current day one competences for veterinary medicine include references to behavioural medicine, a clearly defined set of day one competences is required.

## Additional files

Additional file 1: Questionnaire sent to Private Veterinary Practitioners. (PDF 60 kb)

Additional file 2: Questionnaire sent to Veterinary Nurses. (PDF 60 kb)

## Abbreviations

CCAB: Certified Clinical Animal Behaviourist
ECCVT: The European Coordination Committee for Veterinary Training
FVE: Federation of Veterinarians of Europe
IVNA: Irish Veterinary Nurses Association
PAS: Premises Accreditation Scheme
PDSA: People’s Dispensary for Sick Animals
PVP: Private veterinary practitioner
RVC: Registered Veterinary Clinic
RVH (CA): Companion Animal Registered Veterinary Hospital
VCI: Veterinary Council of Ireland
VN: Veterinary nurses
