# Supplementary material for: Towards the development of day one competences in veterinary behaviour medicine: survey of veterinary professionals experience in companion animal practice in Ireland
Source: Ir Vet J. 2018 May 12;71:12. doi: 10.1186/s13620-018-0123-3 (PMC5948881; doi:10.1186/s13620-018-0123-3)
Supplement: Supplementary file 2 — Questionnaire sent to Veterinary Nurses. (PDF 60 kb) [file 13620_2018_123_MOESM2_ESM.pdf]

Veterinary Nurse Survey on Provision of Animal Behaviour Advice in Clinical Practice

1. Are you a
   Veterinary nurse
   Private veterinary practitioner

2. In what year did you graduate? ________________

3. Where is your practice located?
Urban
Suburban
Rural

4. How many veterinary professionals work in your practice?
Veterinary surgeons
Veterinary nurses

5. What species are treated in your practice? Please tick all that apply.

Cattle
Sheep
Horses
Dogs
Cats
Other *(Please specify)*

6. Do you offer animal behaviour consultations in your practice? (A consultation in which the primary presenting problem is behaviour-related)

Yes
No

7 If you answered Yes to Question 6:
Who provides the behaviour consultations?
Tick all that apply

Veterinary surgeon(s) working in your practice with a qualification in animal behaviour
Veterinary surgeon(s) working in your practice without a qualification in animal behaviour
Veterinary nurse(s) working in your practice with a qualification in animal behaviour
Veterinary nurse(s) working in your practice without a qualification in animal behaviour
An external behaviour specialist who is a veterinary professional
An external behaviour specialist who is not a veterinary professional
Other (*Please specify)*

8. Do you have puppy parties, puppy play dates or other training events in your practice?

Yes
No

9. If you answered Yes to Question 8:
Who provides these events?
Tick all that apply.

Veterinary surgeon(s) working in your practice with a qualification in animal behaviour

Veterinary surgeon(s) working in your practice without a qualification in animal behaviour
Veterinary nurse(s) working in your practice with a qualification in animal behaviour
Veterinary nurse(s) working in your practice without a qualification in animal behaviour
An external behaviour specialist who is a veterinary professional
An external behaviour specialist who is not a veterinary professional
Other (*Please specify)*

10. On average, how often are you asked questions regarding animal behavioural issues when dealing with clients (.e.g in the waiting room, dispensing medication, discharging patients etc.)?

Daily
Weekly
Monthly
Less often

11.From the list below, please select which species you get behavioural queries about. You can select more than one.

Dogs
Cats
Horses
Other, please specify _______________

12. In the last 12 months have animals in your practice been referred to an animal behaviour specialist?

Yes
No

13. If you answered Yes to Question 12, what is the qualification of the behaviour specialist? Tick all that apply.

Veterinary Surgeon with a specialization in behaviour (e.g. MSc, CCAB)
Veterinary Nurse with a specialization in behaviour (e.g. BSc, MSc,)
Certified Clinical Animal Behaviourist (CCAB)
MSc in Companion Animal Behaviour Counselling (MSc CABC)
Member of the Association of Pet Behaviour Counsellors (APBC)
Member of the Association of Pet Dog Trainers (APDT – UK or Ireland)
Member of the Animal Behaviour and Management Alliance (ABMA)
Don't know/Not sure
Other, please specify __________________

14. If you work in small animal practice, how frequently do you encounter the following behavioural issues in dogs in your practice?
Daily
Weekly
Monthly
Less often

Toilet training
Inappropriate elimination
Aggression towards people

Aggression towards other dogs
Dog-reactive (dogs that over-react to other dogs)
Anxiety-related problems (e.g. separation anxiety)
Fearful behaviour (e.g. fear of fireworks, fear of the hoover)
Destructive behaviour (e.g. chewing furniture)
Compulsive disorders (e.g. tail-chasing, acral lick dermatitis)
Unruly behaviour (e.g. pulling on lead, jumping up)
Roaming or escaping
Poor recall
Vocalisation
Other (please specify)

15. If you work in small animal practice, how frequently do you encounter the following behavioural issues in cats in your practice?
Daily
Weekly
Monthly
Less often

House soiling
Destructive behaviour (e.g. scratching furniture)
Aggression towards owner and other family members
Aggression towards guests
Aggression towards other pets
Overgrooming, wool or blanket-sucking
Anxiety-related problems (e.g. hiding)
Vocalisation
Fearful of other cats, dogs or people
Other (please specify)

Q 16 If you work in equine practice, how frequently do you encounter the following behavioural issues?
Daily
Weekly
Monthly
Less often

Handling problems (e.g. headshyness, barging)
Riding problems (e.g. bucking, spooking)
Stereotypies or stable vices (e.g. crib-biting, weaving)

17. What do you consider to be the key challenges to providing animal behaviour consultations? Tick all that apply

Lack of in-house and/or personal expertise
Lack of referral expertise
Clients not willing to pay
Lack of time
Fear of litigation
No demand for animal behaviour consultations
Other (please specify)

18. Which of the following statements best reflect your clinical experience in addressing animal behavioural issues?

I received adequate undergraduate training but do not see a sufficient number of cases to maintain competency
I received adequate undergraduate training and I see a sufficient number of cases to maintain competency
I received adequate undergraduate training and I have obtained a further qualification in veterinary behaviour
I received inadequate undergraduate training to provide clinical advice on animal behaviour
I received inadequate undergraduate training but have developed a competency in animal behaviour
I received inadequate undergraduate training but I have obtained a further qualification in veterinary behaviour
